# Incorporation of Pyranocoumarin, Xanthyletin, in Dental Composite Resins to Produce Biobased Composites: Evaluation of Cytotoxicity and Mechanical and Optical Properties

**DOI:** 10.1155/ijbm/1828509

**Published:** 2026-07-09

**Authors:** Ana M. Herrera-González, A. Lobo Guerrero-Serrano, N. Trejo-Carbajal, J. García-Serrano, M. C. Reyes-Angeles, M. Vargas-Ramírez, G. Ortega-Zarzosa

**Affiliations:** ^1^ Instituto de Ciencias Básicas e Ingeniería, Universidad Autónoma del Estado de Hidalgo, Mineral de la Reforma, Hidalgo, Mexico, uaeh.edu.mx; ^2^ Facultad de Ciencias, Universidad Autónoma de San Luis Potosí, San Luis Potosí, Mexico, uaslp.mx

**Keywords:** composite resin, crosslinking, networks, xanthyletin

## Abstract

Dental organs are naturally fluorescent. In dentistry, composite biomaterials have been sought that match not only the physical and chemical performance of the organs but also the optics so that they have a completely natural appearance. However, matching physical and optical properties has been challenging because materials are required to withstand the tensile forces of dental organs and materials that fluoresce at a specific wavelength. To achieve this, a novel strategy proposed in this work is to use a natural product extracted from the Bloodwood tree, xanthyletin, in dental materials. Xanthyletin, due to its chemical structure, is a natural fluorescent compound capable of dissipating stresses by the formation of hydrogen bonds in a composite resin. The objectives of this study were to extract xanthyletin from the Bloodwood tree and characterize it by NMR and FTIR, as well as to study its fluorescence through the determination of its quantum yield, and subsequently, to evaluate the effect of the addition of xanthyletin on the mechanical, optical, and physicochemical properties in the formulation of dental resin composite (DRC). The results showed that the use of xanthyletin at 0.3% by weight is sufficient to obtain the highest value of flexural strength and elastic modulus, with a statistically significant difference with respect to the control (*p* < 0.05). In addition, due to its fluorescence, xanthyletin, when used in the formulation of composite resins, at a low concentration (0.3%), confers the fluorescence property and improves the natural appearance of composite resins. Double‐bond conversion, polymerization contraction, solubility, and water sorption were not statistically significant compared to the control (*p* > 0.05); however, they met the requirements established by the ISO 4049 Dentistry – Polymer‐Based Restorative Materials standard.

## 1. Introduction

Dental resin composites (DRCs) are materials used in dentistry with great success due to their chemical, mechanical, and optical properties. Change in the elastic modulus (*E*) of DRC is mainly related to the inorganic filler (SiO_2_), while the flexural strength (*σ*) is mainly associated with the organic matrix (crosslink polymer) [1]. Also, the interface between the filler and the organic matrix has a definite effect on the mechanical behavior of DRC [2]. The mechanical properties increase when the silanized inorganic filler is used, due to the strong linkage between the organic matrix and the inorganic filler using a coupling agent that allows dissipating the mechanical energy or the stresses generated in the material [3]. Today, 3‐methacryloxypropyltrimethoxysilane is the predominant coupling agent in dentistry. 3‐Methacryloxypropyltrimethoxysilane has a methacrylate group at one end (which forms C–C bonds with the organic matrix) and methoxysilane groups (which produces –Si–O–Si– bonds with the inorganic filler, SiO_2_) in the other end [4]. However, the Si–O–Si bonds can be vulnerable to hydrolysis reaction because this covalent bond has a significant ionic character due to the electronegativity differences between the atoms (Si–O) [5]. There are reports about the use of other coupling agents with different structures, but conserved the methacrylate and silane end groups; however, the mechanical properties are lower than those obtained with 3‐methacryloxypropyltrimethoxysilane [4].

On the other hand, an important goal in aesthetic restorative dentistry is to design restorations comparable with the optical characteristics of natural teeth. These include color, translucency, opalescence, and fluorescence, which collectively contribute to the essential appearance of a natural tooth [6, 7]. Human teeth are fluorescent, both enamel and dentin are fluorescent tissues. Natural teeth have a blue fluorescence under ultraviolet (UV) light; thus, they appear whiter and lighter under daylight [8, 9]. Studies suggest that fluorescence contributes to the final appearance of aesthetic restorations by providing a visual perception much closer to real teeth [10, 11]. Since the basic components of experimental dental resins are not capable of promoting fluorescence, rare earth inorganic oxide fluorescent species such as europium, cerium, terbium, ytterbium, dysprosium, and/or samarium have been used to enhance this property in dental materials [12–15]. However, the rare earth inorganic oxides are opaque and may reduce the passage of photoactivation light through the composite, thus reducing the curing depth. Bearing this in mind, new approaches must be explored to confer fluorescence to a dental composite.

In nature, there are fluorescent natural products, and among these natural products are coumarins [16, 17]. Xanthyletin is a natural pyranocoumarin that can be extracted from a tree called Bloodwood. Bloodwood is also known as Sanguine Wood due to the release of a red secretion that gives the impression of bleeding wood. Bloodwood (*Brosimum rubescens*) is also known as Granadillo or Mira Piranga [18, 19]. Compounds derived from this tree species are currently used in medicine as a source of biological ingredients due to their bioactivity and pharmacological properties, including antifungal, antibacterial, and antiviral properties [20]. Coumarins have also been used in the manufacture of hybrid composite materials with applications in light‐emitting diodes due to their conjugated π system that facilitates electronic delocalization [21, 22]. However, there are no reports of fluorescent hybrid biomaterials based on organic compounds such as coumarins and pyranocoumarins with dental applications. One of the key objectives of aesthetic restorative dentistry is to create restorations that complement the natural tooth’s optical properties [23]. Color, translucency, opalescence, and fluorescence are optical properties that give the natural tooth its essential appearance [24, 25].

Therefore, taken from the background, the goal of this work is the formulation of experimental composite resins using a natural compound, the xanthyletin, as well as the evaluation of its incorporation in mechanical and optical properties.

## 2. Materials and Methods

All solvents and reagents used in the extraction, characterization of xanthyletin, and formulation of resins were purchased from Sigma‐Aldrich (St. Louis Missouri, MO, USA).

### 2.1. Characterization of the Materials

The structure of the xanthyletin was elucidated by proton nuclear magnetic resonance (^1^H NMR) in a Bruker Ascend^TM^ 400 MHz equipment (Germany). According to the solubility of xanthyletin, deuterated chloroform was used as solvent. The functional groups in the xanthyletin and resin composites were qualitatively identified by Fourier transform infrared spectroscopy (FTIR). The spectra were acquired in mid‐FTIR with attenuated total reflection (ATR) using a PerkinElmer Frontier FTIR (UK). The photopolymerization of composite resins was made with a dental photocuring lamp using a Bluephase 16i (Involaclar‐Vivadent, Liechtenstein, of 800 mW/cm^2^ and wavelength of 430–490 nm). A universal testing machine (Instron model 4465, Norwood, MA) was used to obtain the mechanical properties. UV–vis absorption spectra were measured using a Lambda 35 spectrophotometer, whereas fluorescence spectra were acquired using a PerkinElmer LS55 (UK).

### 2.2. Extraction and Characterization of Xanthyletin

Wood was obtained from the bloodwood tree. A 10‐g piece of wood was ground into fragments measuring approximately 0.5 mm in width and 8 mm in length. The first extraction was carried out as follows: 10 g of ground wood was placed in a beaker with 120 mL of chloroform and stirred constantly at 40°C. The chloroform turned translucent yellow, and the process was repeated three times. The chloroform used in the extraction was then evaporated, yielding a yellow solid with a mass of 0.0984 g. Purification was carried out by recrystallization in chloroform, obtaining xanthyletin as a colorless crystalline solid. The product obtained was xanthyletin, which was characterized by NMR, FTIR, UV–vis, and fluorescence spectroscopy.

 FTIR (KBr) ν_max_ cm^−1^: 1750 νC=O, 1220 and 1158 νC–O–C; 1622, 1500 νC=C_aromatic_, 2974, 2926, 2846 νCH).
 
^1^H RMN (*δ*, CDCl_3_, 400 MHz): 1.43 (6H, s, H10), 5.86 (1H, d, *J* = 8.0 Hz, H7), 6.27 (1H, d, *J* = 8.0 Hz, H3), 6.49 (1H, d, *J* = 12.0 Hz, H6), 6.77 (1H, s, H9), 7.42 (1H, s, H5) and 7.92 (1H, d, *J* = 12.0 Hz, H4).

### 2.3. Determination of Fluorescence Quantum Yield of Xanthyletin

To determine the fluorescence quantum yield (Φ*x*) of xanthyletin, curcumin was used as standard and ethanol as solvent. The Φ*x* was determined using equation (1) according to the procedure reported in the literature [26].

(1) Φx=ΦSTGradxGradSTηx2ηST2,

where Φ*x* is the quantum yield of the sample, Φ_ST_ is the quantum yield of the standard, Grad_*x*_ and Grad_ST_ represent the obtained gradients of fluorescence intensity as a function of the absorbance of the samples and the standard, respectively, and *η*_*x*_ and *η*_ST_ are the refractive indices of the solvents used to prepare the solutions of the test samples and the standard, respectively.

### 2.4. Formulation of Composite Resin

The composite resins intended for experimentation were prepared by combining BisGMA and TEGDMA as organic matrix, in two ratios of 65/35 and 70/30 wt.%, respectively. The initiator consisted of both camphorquinone (CQ) and 4‐diethylaminobenzoate (EDAB) at concentrations of 0.5 and 1 wt.%, respectively, in all the composites. First, the initiator system was added to the BisGMA/TEGDMA mixture. Then, the unsilanized or silanized SiO_2_ filler, 5 mL of ethanol, and xanthyletin at concentrations of 0.2, 0.3, and 0.5 wt.% were placed in a beaker and mixed for 30 min, after mixing, the ethanol was evaporated. Finally, the SiO_2_/xanthyletin filler was incorporated into the BisGMA/TEGDMA mixture (organic matrix) to obtain the final DRCs. A control group was also prepared without the addition of xanthyletin, Table 1.

**TABLE 1:** Formulation of composite resins.

Composite	SiO_2_	Xanthyletin	BisGMA	TEGDMA
	*Filler 65 wt.%*		*Organic matrix 35 wt.%*	
Control unsilanized 65/35	100	0	70	30
Control silanized 65/35	100	0	70	30
Experimental resin 1	99.8	0.2	70	30
Experimental resin 2	99.7	0.3	70	30
Experimental resin 3	99.5	0.5	70	30

	*Filler 70 wt.%*		*Organic matrix 30 wt.%*	
Control unsilanized 70/30	100	0	70	30
Control silanized 70/30	100	0	70	30
Experimental resin 4	99.8	0.2	70	30
Experimental resin 5	99.7	0.3	70	30
Experimental resin 6	99.5	0.5	70	30

*Note:* The photoinitiator system was the same in all resins camphorquinone/EDAB (0.5/1 wt.%).

### 2.5. Flexural Strength and Elastic Modulus

The flexural strength and elastic modulus were assessed according to ISO 4049 International Standard [27]. The dimensions of the specimens, 25 mm × 2 mm × 2 mm (*n* = 7), were measured using a digital calibrator with a precision of 0.01 mm. The flexural strength and elastic modulus were calculated according to the formulas specified in the ISO standard.

### 2.6. Polymerization Kinetics and Double‐Bond Conversion (DC)

The polymerization kinetics and degree of DC were evaluated using FTIR with the ATR technique according to the report in the literature [28, 29]. The experiment was performed three times for each sample (*n* = 3). The absorption bands used in this experiment were the absorption band of aliphatic νC=C bond at 1636 cm^−1^ and the absorption band of the aromatic νC=C bond at 1611 cm^−1^.

### 2.7. Polymerization Shrinkage

Polymerization shrinkage was calculated following the ISO 17304 standard [30]. The weight of small samples (*n* = 6) of the unpolymerized and polymerized material from each group were weighed using an analytical balance with a density determination kit. The measurements were performed at controlled temperatures in a room devoid of humidity.

### 2.8. Optical Fluorescence Analysis of Composite Resins

In this test, cylindrical samples measuring 4 × 1 mm (*n* = 3) were used. This experiment was made using a black box with dimensions of 45 cm in height, length, and depth. The samples were positioned inside the box at 10 cm of light source. To serve as the light source, a UV LED medium‐beam lamp (UltraFire WF‐ 501B, Arizona Tactical Gear, Phoenix, AZ, USA) with a wavelength of 385 nm was placed inside the box. On top of the box, a Nikon D5000 DSLR camera, equipped with a 100 mm Macro lens was mounted. Photographs were analyzed using Photoshop CC 14.0 image software. During the analysis, the average lightness (*L*) component based on the CIE lab color space was recorded [15].

### 2.9. Cell Viability Assay

Cell viability of the experimental resins containing xanthyletin was assessed using the 3‐(4,5‐dimethylthiazol‐2‐yl)‐2,5‐diphenyltetrazolium bromide (MTT) assay. Samples of the experimental and control resins were prepared in the form of discs with a diameter of 5 mm (*n* = 6). Prior to the assay, a cell density of 7.500 cells per well was seeded in 96‐well flat‐bottom plates with a volume of 100 μL of Dulbecco’s modified Eagle medium (DMEM) per well. They were subsequently incubated for 24 h (37°C with 5% CO_2_).

The resin samples were placed in 24‐well plates with 1 mL of DMEM and stored at 37°C at pH 7. After 24 h, 200 μL of the eluate from each resin was transferred to the plate. The 96‐well plates containing the precultured cells were then incubated under the same conditions for 24 h. After this time, the culture medium was removed, and 100 μL of new culture medium and 20 μL of MTT (5 mg/50 mL of PBS) were added. The plates were incubated for 4 h (37°C with 5% CO_2_). Finally, the supernatant was removed and 100 μL of DMSO was added. The culture plates were measured using a photometer at the MTT wavelength of 570 nm (Thermo Scientific Multiskan FC). Percent cell viability values were calculated using the following equation:

(2) cell viability %=absorbance of treated cellsabsorbance of control cells×100.

### 2.10. Statistical Analysis

Statistical analysis was performed using Sigma Plot 12.0 software. The data were analyzed to test the requirement of normal distribution and the homogeneity of variance. A *t*‐test (Student’s *t*) was performed to detect statistically significant differences among the groups. In all cases, the significance value was set to *α* > 0.05.

## 3. Results and Discussion

### 3.1. Xanthyletin Characterization: FTIR and NMR

**FIGURE 1:**
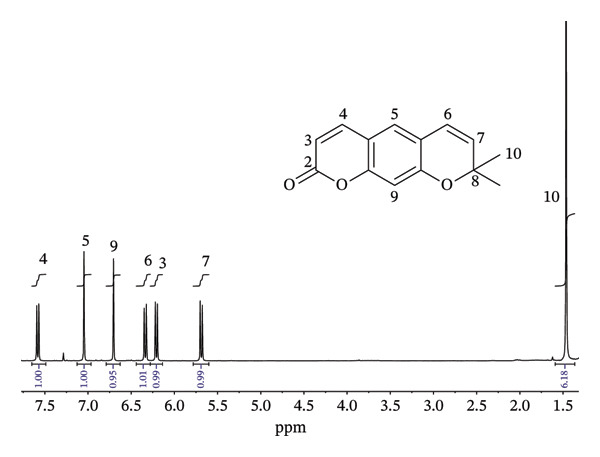
^1^H NMR spectrum of xanthyletin in CDCl_3_, 400 MHz.

**FIGURE 2:**
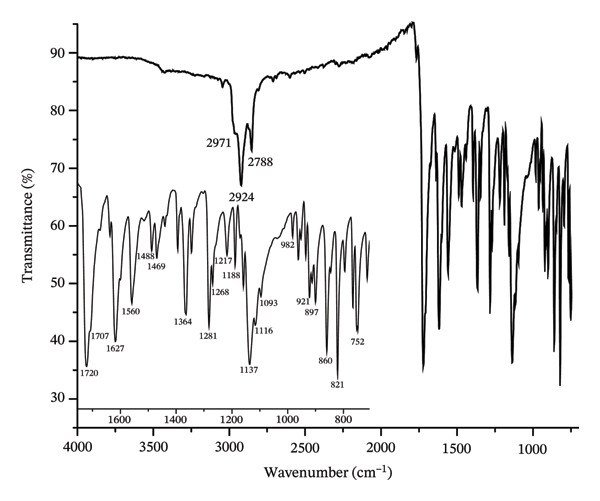
FTIR spectrum of xanthyletin and magnified view of the 1800 to 700 cm^−1^ regions.

Xanthyletin was extracted from the tree bloodwood in chloroform and obtained spectroscopically pure after recrystallization. The obtained product was a colorless solid. Of the total wood weight, 0.98% corresponds to xanthyletin. Its structure was confirmed by ^1^H NMR and FTIR spectroscopy, as shown in Figures 1 and 2.

The ^1^H NMR spectrum shows seven signals that correspond to the types of protons present in xanthyletin (Figure 1). The sum of all the signals in the spectrum integrates for 12 protons, consistent with the compound’s structure. Signals from 5.5 to 8 ppm correspond to the six aromatic protons, while the signal at the lower frequency of 1.43 ppm corresponds to the six protons from the two methyl groups present in the xanthyletin structure [20, 21].

Xanthyletin is a pyranocoumarin, which is an aromatic compound characterized by containing an ester and an ether group in its structure. Therefore, the main absorption bands by FTIR spectroscopy, which provide evidence of xanthyletin extraction, are the three characteristic absorption bands of the ester group at 1720, 1281, and 1137 cm^−1^, corresponding to the vibration modes νC=O, ν_asymmetric_C‐O, and ν_symmetric_C–O, respectively. The absorption bands at 1627 and 1560 cm^−1^ were assigned to νC=C of the aromatic ring, while the absorption bands at 2971 and 821 cm^−1^ were attributed to the vibration modes νC–H and δC–H out of the plane of the aromatic ring, respectively. The three characteristic absorption bands of the ether group were found at 1268 cm^−1^ for ν_asymmetric_ = C–O–C, 1093 cm^−1^ for ν_symmetric_ = C–O–C, and 860 cm^−1^ for νC–O. Finally, the absorption bands at 2924 and 2788 cm^−1^ were assigned to νC–H of the methyl groups, and at 1448 and 1364 cm^−1^, absorption bands were observed due to the deformation of the methyl groups (δC–H), Figure 2.

### 3.2. Quantum Yield of Xanthyletin

**FIGURE 3:**
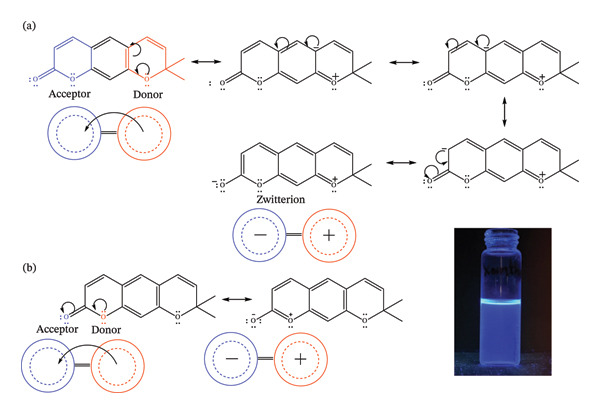
Resonance structures of xanthyletin, incanting push–pull system, and picture of solution of xanthyletin (0.01 mg/5 mL) in CHCl_3_.

Xanthyletin is a fluorescent natural compound. Table 2 summarizes its fluorescence parameters, including the absorption wavelength (*λ*), emission wavelength (*λ*_em_), excitation wavelength (*λ*_ex_), molar extinction coefficient (*ε*), Stokes shift (Δ*λ*), and quantum yield (*φx*) of xanthyletin and the standard compound. Organic molecules with a delocalized *π*‐electron system are attractive targets for applications in advanced functional materials [31]. Closed organic systems with an electron donor (D) and an electron acceptor (A) represent a class of π‐conjugated systems known as push–pull systems [32]. Due to its structure, xanthyletin can be considered a push–pull system with intramolecular charge transfer, as shown in Figure 3, which induces the highest fluorescence. Two possible pathways of resonance structures could be expressed (Figure 3).

**TABLE 2:** Spectroscopic properties and quantum yield of xanthyletin.

	*λ*_ex_ (nm)	*ε* (cm^−1^·mol^−1^)	*λ*_onset_ (nm)	Eg (eV)	*λ*_em_ (nm)	Δ*λ* (nm)	*φx*
Curcumin	428	5176	492	2.520	523	95	0.063
Xanthyletin	351	12455	391	3.171	409	58	0.316

Both pathways produce π‐conjugated zwitterions. Both zwitterions are preferentially represented in terms of an electron donor moiety bearing a net negative net charge, and moiety electron acceptor one bearing a positive net charge connected by various π‐conjugated bridges [32]. Coumarin derivatives due to π‐conjugated system are fluorescent materials [16]; therefore, xanthyletin could be useful as fluorescent material in composite resins.

The *ε* value for xanthyletin is higher than the standard (curcumin), indicating its strong photon absorption (Table 2). It has been reported that dentin fluorescence is caused by several organic compounds, but this autofluorescence remains controversial and continues to be studied. Several luminescent compounds have been proposed in the literature as responsible for fluorescence in dental tissues, such as pyrimidine, tryptophan, tyrosine, hydroxy pyridine, pyrinoline, and hydroxyapatite complexes with pyrinoline. These compounds have emission bands in the range of 340–400 nm, while dentin emits at 400 nm. Xanthyletin is a compound with a conjugated π‐electron system, making it an attractive fluorescent organic compound with an emission band being at 409 nm. The experimental resins formulated with xanthyletin exhibit fluorescence properties in the range of compounds found in dental tissues, because the *λ*_em_ of xanthyletin is at 409 nm.

Finally, the difference between the spectral positions of *λ*_ex_ and *λ*_em_ represents a measure of the energy conversion efficiency between the processes of photon absorption and emission in a molecule. It is desirable for materials intended for luminescent applications to have low Stokes shift values (Δ*λ*). Xanthyletin exhibited the smallest value, and therefore, it achieved the highest quantum yield. Due to its high quantum yield, xanthyletin requires very low concentrations within composite material formulations to confer the property of fluorescence (0.3 wt.%).

### 3.3. Composite Resin Characterization

#### 3.3.1. FTIR Characterization of the Xanthyletin–SiO_2_ Mixture

**FIGURE 4:**
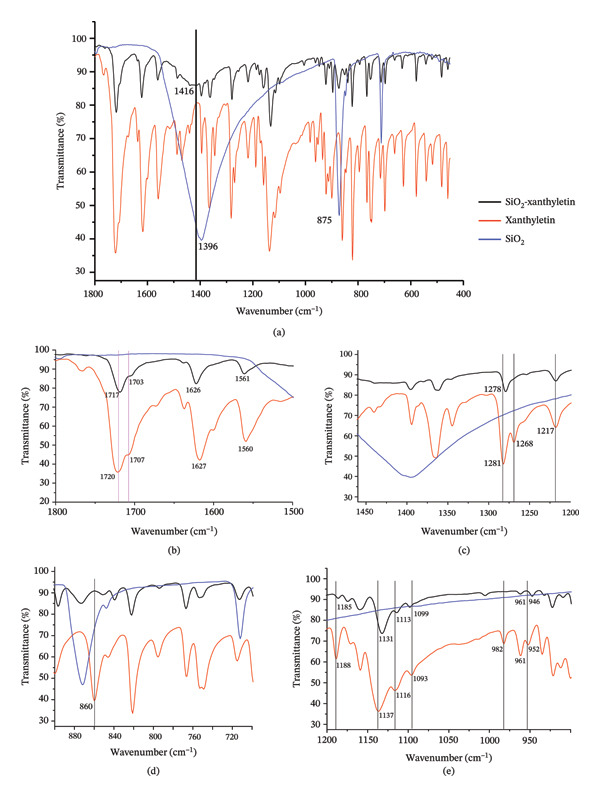
FTIR spectra of xanthyletin, xanthyletin–SiO_2_ mixture, and SiO_2_ (a), magnifications in different wavenumber regions (b–e).

The trees are mainly composed of cellulose, lignin, and hemicellulose. Also, in smaller quantities, they contain resins, tannins, starch, oils, or organic compounds such as xanthyletin, depending on the type of tree. All these components in the tree form a composite material that interacts through molecular interactions such as hydrogen bonds [33, 34]. It has been reported that cellulose through its hydroxyl groups and oxygen atoms forms inter and intramolecular hydrogen bonds with itself [35, 36] or with other molecules forming composite materials [37, 38] strongly increasing mechanical properties [39–41]. SiO_2_, although in much smaller proportion than cellulose, has hydroxyl groups and forms hydrogen bonds with the oxygen atoms of xanthyletin when these are combined. Figure 4(a) shows three overlapping FTIR spectra: xanthyletin, the xanthyletin–SiO_2_ mixture, and SiO_2_. Additionally, to more easily observe the wavenumbers of the absorption bands, magnified FTIR spectra are shown (Figures 4(b–e)). When the FTIR spectrum of the xanthyletin–SiO_2_ mixture is compared with that of SiO_2_, a shift of the νSi–O absorption band to higher wavenumbers can be clearly observed. This band is located at 1396 cm^−1^ in SiO_2_ and at 1416 cm^−1^ in the xanthyletin–SiO_2_ mixture. The change is due to the formation of hydrogen bonds between the oxygens of the ester and ether groups of xanthyletin and the hydroxyl groups of SiO_2_. This change in wavenumber is also observed in some absorption bands of xanthyletin, mainly in the bands associated with the ester and ether groups, corroborating the previous statement.

**FIGURE 5:**
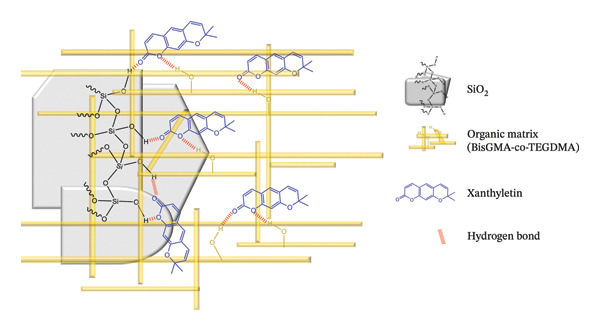
Intermolecular hydrogen bonding between SiO_2_, xanthyletin, and the organic matrix.

The characteristic absorption bands of the ester group in xanthyletin at 1720, 1281, and 1137 cm^−1^ due to the νC=O, ν_asymmetric_C–O, and ν_symmetric_C–O vibration modes, respectively, change in the xanthyletin–SiO_2_ mixture to 1717, 1278, and 1131 cm^−1^. The three absorption bands in the mixture are shifted to lower wavenumbers because the lone pairs of electrons on the oxygen atoms form dipole–dipole interactions and hydrogen bonds, preventing their electronic delocalization across the three fused rings as described in Figure 5 [42]. The three characteristic absorption bands of the ether group in xanthyletin were found at 1268 cm^−1^ ν_asymmetric_C–O–C, 1093 cm^−1^ ν_symmetric_C–O–C, and 860 cm^−1^ νC–O, which also have changes in the xanthyletin–SiO_2_ mixture. In the SiO_2_–xanthyletin mixture, the absorption bands at 1268 cm^−1^ and 860 cm^−1^ considerably decrease their percentage in transmittance, while the absorption band at 1093 cm^−1^ shifts to 1099 cm^−1^, indicating the formation of dipole–dipole interactions and hydrogen bonds.

#### 3.3.2. Evaluation of Mechanical Properties: Flexural Strength and Elastic Modulus

To determine the influence of xanthyletin on the flexural strength and elastic modulus of the resins, four control resins and six experimental resins were formulated with different concentrations of xanthyletin. Table 3 shows the results of flexural strength and elastic modulus using 0.2, 0.3, and 0.5 wt.% of xanthyletin. It was observed that increasing the concentration of xanthyletin in experimental materials 1, 2, 4, and 5 increased flexural strength and elastic modulus. However, a decrease was observed in experimental resins 3 and 6.

**TABLE 3:** Flexural strength and elastic modulus of composites.

Composite	Xanthyletin wt %	Flexural strength (MPa)	Elastic modulus (GPa)
*Filler/organic matrix 65/35*			
Control unsilanized 65/35	0	35.1 (1.0)^a^	3.4 (1.6)^a^
Control silanized 65/35	0	41.3 (0.9)^b^	5.6 (1.8)^b^
Experimental resin 1	0.2	45.6 (2.5)^b^	5.5 (5.4)^b^
Experimental resin 2	0.3	58.7 (1.4)^c^	8.7 (5.3)^c^
Experimental resin 3	0.5	43.6 (6.4)^b^	4.6 (6.6)^b^

*Filler/organic matrix 70/30*			
Control unsilanized 70/30	0	45.0 (2.6)^a^	5.3 (4.8)^a^
Control silanized 70/30	0	45.4 (2.2)^a^	6.4 (5.8)^a^
Experimental resin 4	0.2	42.6 (2.2)^a^	6.6 (2.8)^a^
Experimental resin 5	0.3	51.6 (1.8)^b^	9.3 (1.1)^b^
Experimental resin 6	0.5	41.1 (2.1)^a^	8.6 (2.7)^b^

*Note:* Different lowercase letters in each column indicate the presence of statistically significant differences (*p* < 0.05).

The new resins must exhibit mechanical properties equal to or greater than those of the controls to ensure acceptable clinical performance. The experimental resins that showed the highest values of flexural strength and elastic modulus were those with a concentration of 0.3 wt.% of xanthyletin and therefore showed a statistically significant difference with the control resins (*p* < 0.05). This may be because 0.3 wt.% is the optimal concentration to generate the highest number of interactions between xanthyletin, the organic matrix, and the inorganic filler (SiO_2_) through intermolecular forces such as hydrogen bonds (Figure 5), and therefore, xanthyletin acts as a coupling agent, enhancing both properties. However, at a concentration of 0.2 wt.% xanthyletin, the hydrogen bond is not enough to produce a statistically significant increase in both properties compared with the controls. On the other hand, when xanthyletin is present in excess (0.5 wt.%), its spontaneous radiation absorption prevents the passage of light through the resin. This can negatively affect the degree of DC, resulting in a decrease in mechanical properties.

At low concentrations, xanthyletin enhances mechanical and optical properties. However, one of the limitations was the purification of xanthyletin. The first technique used for its purification was silica gel (SiO_2_) column chromatography using CHCl_3_ as the mobile phase [43]. However, due to hydrogen bond formation, the recovery of pure xanthyletin was only 10% of the initial mass. Therefore, a recrystallization technique in CHCl_3_ was used, from which colorless crystals were obtained with a 92% recovery of the initial mass [44]. It was found, as a limitation, that this technique requires slow evaporation of the solvent. Also, during the formulation of the experimental resins, an analytical precision balance was required to weigh the xanthyletin, since any increase or decrease in mass significantly affects the properties. Therefore, it was necessary to repeat the experiments for each test (flexural strength, fluorescence, solubility, and water sorption) at least three times to ensure reproducibility.

#### 3.3.3. Depth of Cure

After mechanical evaluation, it was determined that the best resins were those formulated with 0.3 wt.% xanthyletin in both 65/35 and 70/30 SiO_2_/organic matrix systems. Table 4 shows the results of the depth of cure for experimental resins 2 and 5 and their respective controls. The depth of cure of experimental resins did not show statistically significant differences compared to the controls.

**TABLE 4:** Curing depth of experimental and control resins.

Composite	Xanthyletin wt %	Filler/organic matrix 65/35	Filler/organic matrix 70/30
		Depth of cure	Depth of cure
Control silanized 65/35	0	2.16 (0.11)^a^	
Experimental resin 2	0.3	1.99 (0.08)^a^	
Control silanized 70/30	0		2.27 (0.33)^a^
Experimental resin 5	0.3		2.09 (0.06)^a^

*Note:* Different lowercase letters in each column indicate the presence of statistically significant differences (*p* < 0.05).

The depth of cure reflects the effectiveness of the polymerization process, thereby minimizing the risk of unpolymerized monomers leaching into the oral cavity and reducing microleakage. After mechanical evaluation, it was determined that the best resins were those formulated with 0.3 wt.% xanthyletin in both 65/35 and 70/30 SiO_2_/organic matrix systems. In Table 4, it is evident that there are no statistically significant differences between the depth of cure of the control and experimental resins 2 and 5. However, the flexural strength and elastic modulus values of the experimental resins show a statistically significant increase. In other words, xanthyletin improves the mechanical properties and provides fluorescence to the dental resins without altering the polymerization reaction at low concentrations.

**FIGURE 6:**
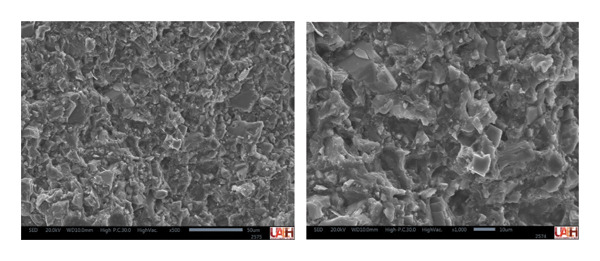
SEM image of experimental resin 2 after the fracture at × 500 and × 1000.

To demonstrate the homogeneous distribution of the filler within the organic matrix in the experimental specimens, Experimental resin 2 was analyzed by scanning electron microscopy (SEM) after fracture (Figure 6). In the micrographs, SiO_2_ particles with heterogeneous shapes can be observed. However, their distribution within the organic matrix is homogeneous. In addition, due to the fracture, surface roughness corresponding to the size of the SiO_2_ particles can be observed.

#### 3.3.4. Polymerization Kinetics

**FIGURE 7:**
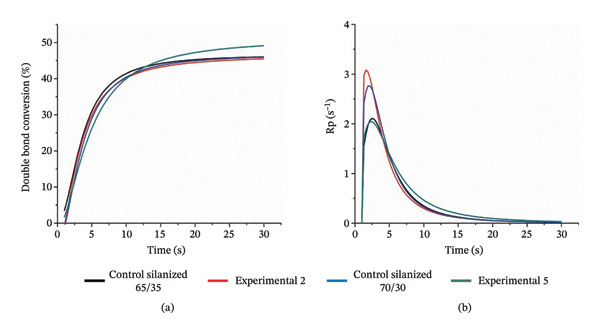
Polymerization kinetics of the evaluated materials: (a) Degree of conversion and (b) polymerization rate.

Polymerization kinetics includes the evaluation of the degree of DC and the polymerization rate. The results of DC and polymerization kinetics are shown in Figure 7. According to the DC analysis (Figure 7(a)), the differences between the control and experimental groups for each formulation were not statistically significant (65/30, *p* = 0.642; 70/30, *p* = 0.372). However, experimental resin 5 showed a lower polymerization rate.

The analysis of DC and polymerization kinetics demonstrates that the addition of xanthyletin (0.3 wt.%) does not interfere with the photopolymerization reaction of the organic matrix (Figure 7). It has been reported that higher DC increases flexural strength and elastic modulus; however, surprisingly, the DC does not present significant differences with respect to the controls, but flexural strength and elastic modulus increase and show statistical differences. This can be explained by the fact that xanthyletin forms strong dipole–dipole intermolecular interactions with SiO_2_ and the organic matrix, which causes a statistically significant increase in flexural strength and elastic modulus without being part of the photopolymerization reaction. In the analysis of the polymerization rate (Figure 7(b)), a considerable increase in this property is observed for experimental resin 2. This increase may be mainly due to the viscosity of this formulation, since when the TEGDMA monomer is present in a higher concentration, the viscosity of the material decreases and, therefore, the ease of diffusion and propagation of the polymer chains in the polymerization increases. On the other hand, when analyzing the control formulations against the experimental ones, it is possible to observe a slight decrease in the polymerization rate in experimental resin 5. This may be because xanthyletin, by promoting the formation of secondary hydrogen bonds, hinders the mobility of the growing chains during the polymerization reaction, decreasing the rate of the polymerization reaction of the material.

#### 3.3.5. Volumetric Shrinkage

Table 5 shows the volumetric shrinkage values for the groups tested. According to the analysis of volumetric shrinkage, experimental resin 2 showed a statistically significant increase in volumetric shrinkage (*p* ≤ 0.019). This result suggests that xanthyletin promotes greater volumetric shrinkage in the resins. This could be because xanthyletin forms hydrogen bonds with the organic matrix and the inorganic filler, which causes greater packing due to the decrease in free volume between the polymer networks; also, this experimental resin has a lower concentration of SiO_2_ (65 wt.%). On the other hand, experimental resin 5 has no statistically significant difference with the control resin, likely due to the higher amount of inorganic filler (70 wt.%) used.

**TABLE 5:** Water sorption and solubility of the materials evaluated.

Group	Water sorption	Solubility	Volumetric shrinkage
Control silanized 65/35	14.77 (5.22)^a^	4.63 (3.52)^a^	5.95 (0.75)^a^
Experimental resin 2	14.43 (3.50)^a^	3.99 (2.35)^a^	10.49 (0.69)^b^
Control silanized 70/30	16.42 (3.2)^a^	4.74 (3.1)^a^	5.97 (0.74)^a^
Experimental resin 5	16.84 (4.7)^a^	3.88 (1.47)^a^	6.16 (0.52)^a^

*Note:* Equal lowercase letters indicate the absence of statistically significant differences.

#### 3.3.6. Water Sorption and Solubility

The results of water sorption and solubility tests are summarized in Table 5. The analysis determined that there were no statistically significant differences between the control and experimental groups in either the water sorption or solubility.

It is important to note that water sorption of all the materials evaluated was within the limits permitted by ISO 4049. Water sorption is an inherent property of all dental composite materials. In these systems, a certain degree of water uptake is desirable, as it compensates for the volumetric shrinkage that occurs during polymerization. Consequently, related effects such as tooth sensitivity and microleakage are mitigated in materials capable of absorbing water. Furthermore, the absence of a statistically significant difference between the control and experimental groups demonstrated that xanthyletin achieves the formation of stable interactions between the organic matrix and the inorganic filler.

#### 3.3.7. Resin Fluorescence

**FIGURE 8:**
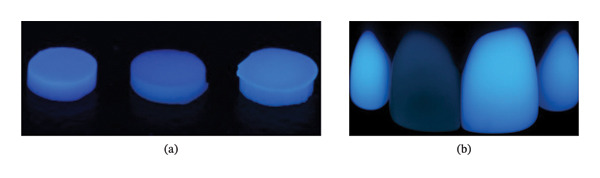
Photograph of the resins under UV–vis light from left to right: experimental resin 5, control, and Filtek Z250 (a) and image of the teeth without fluorescent resin (b).

Figure 8 shows a photograph of the resins from which fluorescence values were obtained. The resins were used in the following order: experimental resin 5, control, and commercial resin (Filtek Z250, 3M ESPE). For this test, no statistically significant differences were observed between experimental resin 5 and the commercial resin (*p* < 0.001); however, the highest fluorescence value recorded was for experimental resin 5 formulated with xanthyletin (Table 6). On the other hand, statistically significant differences were found between experimental resin 5 and the control resin, since the control resin does not contain any fluorescent compounds, with the highest value observed for experimental resin 5 formulated with xanthyletin (Table 6). Therefore, xanthyletin is a fluorescent, biobased compound that can confer fluorescence to dental resins at very low concentrations (0.3 wt.%) while matching the fluorescence properties of a commercial resin.

**TABLE 6:** Resin fluorescence.

Resin	Fluorescence u.a
Experimental resin 5	101.7 (0.02)^a^
Control	88.5 (0.04)^b^
Filtek Z250	98.5 (0.01)^a^

*Note:* Different lowercase letters indicate statistically significant differences (*p* < 0.05).

#### 3.3.8. Cell Viability

**FIGURE 9:**
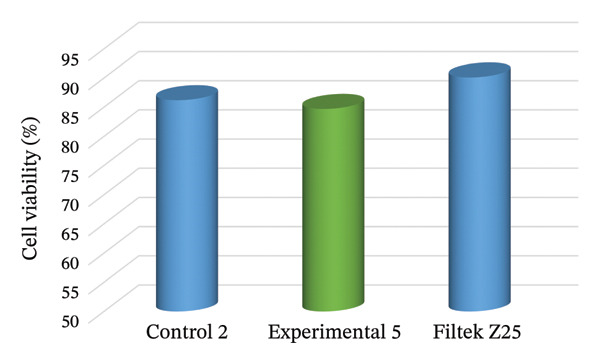
Cell viability of experimental and control resin composites.

The results in Figure 9 show the effects of the eluate obtained from resins on the viability of L929 mouse fibroblast cells. Statistical analysis of the viability of L929 mouse fibroblast cells revealed no significant differences among the tested resins (*p* > 0.05) (Figure 9). Both the control resin and experimental resin 5 demonstrated a cell viability above 85%, indicating the absence of cytotoxicity toward mouse fibroblast cells. The lack of cytotoxic effects could be attributed to the relatively high degree of DC observed in the materials (approximately 60%), and the xanthyletin remains in the resins through the hydrogen bond. Therefore, it is expected that the presence of unreacted monomers is minimal, posing no significant concerns [45], and consequently, experimental resin 5 has potential application as a dental biomaterial.

Due to the excellent results shown by the experimental resins formulated with the natural pyranocoumarin xanthyletin, future work will focus on designing and studying monomers containing coumarins or pyranocoumarins in their structure that can now be covalently bonded.

## 4. Conclusions

Xanthyletin at low concentrations increases the elastic modulus and flexural strength, acting as a coupling agent. Therefore, it can replace the use of organosilanes as coupling agents in dental resins and suppress the chemical reaction between the organosilane and SiO_2_. The use of xanthyletin as a coupling agent is an alternative to the silanization process because it does not require a chemical reaction like the one that occurs between the organosilane and SiO_2_. Furthermore, xanthyletin is a natural, antibacterial product, and its use promotes the production of biobased resins. Finally, due to its chemical structure, xanthyletin in π‐conjugated systems or push–pull systems is a fluorescent compound, and when it is used in the formulation of dental resins, at a low concentration (0.3 wt.%), it confers the fluorescent property and enhances the natural appearance of dental resins.

## Funding

This work was supported by Mexican Secretariat of Science, Humanities, Technology, and Innovation (SECIHTI), project number CBF‐2025‐I‐2357.

## Conflicts of Interest

The authors declare no conflicts of interest.

## Data Availability

The data that support the findings of this study are available from the corresponding author upon reasonable request.
